# Interactions Between *Ephedra sinica* and *Prunus armeniaca*: From Stereoselectivity to Deamination as a Metabolic Detoxification Mechanism of Amygdalin

**DOI:** 10.3389/fphar.2021.744624

**Published:** 2021-11-26

**Authors:** Yan Qin, Shanshan Wang, Qiuyu Wen, Quan Xia, Sheng Wang, Guanjun Chen, Jiayin Sun, Chenlin Shen, Shuai Song

**Affiliations:** ^1^ Inflammation and Immune Mediated Diseases Laboratory of Anhui Province, School of Pharmacy, Anhui Institute of Innovative Drugs, Institute for Liver Diseases of Anhui Medical University, Anhui Medical University, Hefei, China; ^2^ Department of Pharmacy, The First Affiliated Hospital of Anhui Medical University, Hefei, China; ^3^ The Grade 3 Pharmaceutical Chemistry Laboratory of State Administration of Traditional Chinese Medicine, Hefei, China; ^4^ Center for Scientific Research of Anhui Medical University, Hefei, China; ^5^ Hefei Kaifan Analytical Technology Co., Ltd., Hefei, China

**Keywords:** amygdalin, LC-MS/MS, pharmacokinetic, stereoselectivity, compatibility, metabolism, detoxification

## Abstract

Mahuang–Xingren (MX, *Ephedra sinica* Stapf-*Prunus armeniaca* L.) is a classic herb pair used in traditional Chinese medicine. This combined preparation reduces the toxicity of Xingren through the stereoselective metabolism of its *main active ingredient* amygdalin. However, whether stereoselectivity is important in the pharmacokinetic properties of amygdalin either in the traditional decoction or in the dispensing granules is unclear. Amygdalin is hydrolyzed to its metabolite, prunasin, which produces hydrogen cyanide by degradation of the cyano group. A comprehensive study of the metabolic pathway of amygdalin is essential to better understand the detoxification process. In this article, the potential detoxification pathway of MX is further discussed with regard to herb interactions. In this study, the pharmacokinetic parameters and metabolism of amygdalin and prunasin were investigated by comparing the traditional decoction and the dispensing granule preparations. In addition, several potential metabolites were characterized in an incubation system with rat liver microsomes or gut microbial enzymes. The combination of Xingren with Mahuang reduces exposure to *D*-amygdalin *in vivo* and contributes to its detoxification, a process that can be further facilitated in the traditional decoction. From the *in vitro* co-incubation model, 15 metabolites were identified and classified into cyanogenesis and non-cyanogenesis metabolic pathways, and of these, 10 metabolites were described for the first time. The level of detoxified metabolites in the MX traditional decoction was higher than that in the dispensing granules. The metabolism of amygdalin by the gut microbial enzymes occurred more rapidly than that by the rat liver microsomes. These results indicated that combined boiling both herbs during the preparation of the traditional decoction may induce several chemical changes that will influence drug metabolism *in vivo*. The gut microbiota may play a critical role in amygdalin metabolism. In conclusion, detoxification of MX may result 1) during the preparation of the decoction, in the boiling phase, and 2) from the metabolic pathways activated *in vivo*. Stereoselective pharmacokinetics and deamination metabolism have been proposed as the detoxification pathway underlying the compatibility of MX. Metabolic detoxification of amygdalin was quite different between the two combinations, which indicates that the MX decoctions should not be completely replaced by their dispensing granules.

## Introduction

The combination of *Ephedra sinica* Stapf (Mahuang) and *Prunus armeniaca* L. (Xingren) is a classical herb combination used to treat cold-induced asthma in traditional Chinese medicine (TCM) (Ma et al., 2021). According to the *Guidance for Corona Virus Disease 2019: Prevention, Control, Diagnosis and Management* published in China, MX can be combined with other herbs, such as *Glycyrrhiza uralensis* Fisch. (Gancao), *Astragalus mongholicus* Bunge (Huangqi), and *Atractylodes lancea* (Thunb.) DC. (Cangzhu) has been shown to be effective against the new coronavirus and has significantly reduced the proportion of patients with mild symptoms transforming into more severe cases (Zhao et al., 2021a; Zhang et al., 2021). Ephedrines in Mahuang and amygdalin of Xingren have been considered as the main active constituents possessing anti-asthmatic (Wang et al., 2021), anti-inflammatory (Karimi et al., 2021), and antitussive properties (Wang et al., 2019). The main active ingredient of Xingren is amygdalin (3% in Xingren) (Zhang et al., 2020). Amygdalin is metabolized by β-glucosidase, found in plants or the animal gut, into glucose and prunasin. In turn, prunasin, a cyanogenic diglycoside, is metabolized into glucose and mandelonitrile, which are further hydrolyzed to benzaldehyde and hydrogen cyanide. Hydrogen cyanide is rapidly absorbed by the gastrointestinal tract and is distributed throughout the body by the blood circulation after absorption. Hydrogen cyanide is responsible for the antitussive properties of Xingren. However, the cyanogenesis metabolism pathway results in the release of hydrogen cyanide after oral administration of amygdalin and is a well-known mechanism for metabolic poisoning (Cressey and Reeve, 2019).

The essential and common characteristics of TCM preparations are defined by specific formulations that are developed in accordance with the compatibility theory. Different herbal preparations are combined to obtain multi-herb drug cocktails of TCM (Hu et al., 2005). The underlying principle of these formulations involves not only a simple mixture of botanical drugs but also processes able to reduce toxicity and increase treatment efficiency. The MX decoction is composed of Mahuang and Xingren, at a 1:1 dosage combination as described in the “Treatise on Cold Pathogenic and Miscellaneous Diseases.” MX was initially included in Shennong’s Classic of Materia Medica and ranked as a medium grade drug, which means it can present intermediate toxic effects (Dai et al., 2019). Some adverse reactions and even fatal poisoning have been reported in the literature (Blaheta et al., 2016; Han et al., 2018). In our previous study (Song et al., 2016), the contents and pharmacokinetics of alkaloids such as ephedrine and methylephedrine in MX were studied. Mahuang antagonizes the acute toxicity of Xingren and significantly improves the safety profile of the MX combination. Stereoselective pharmacokinetics and cytotoxicity data indicate that the antagonism properties of *D*-amygdalin metabolism may contribute to its detoxification. Whether the stereoselectivity of amygdalin pharmacokinetics depends on the traditional decoction or on the combination of the individual herbs is still unclear.

The traditional decoction is the most common form of administration of TCM (Wang et al., 2018). Herbal materials are often extracted with water to form an aqueous extract (Wang et al., 2012). Dispensing granules, consisting of a single herb preparation in a single formula, have become increasingly popular in the TCM clinic. The granules not only share the benefits of a customized formulation but also allow convenient storage and quality control (Denesh et al., 2021). However, it is not clear whether stereoselective pharmacokinetics are relevant in MX dispensing granule preparation as there is still discussion on the metabolic pathways of amygdalin metabolism *in vivo*. Amygdalin is a cyanogenic glucoside naturally produced by plants (Kovacikova et al., 2019). Metabolic detoxification refers to the process of reducing exposure to harmful xenobiotics *in vivo* which could also contribute to its pharmacokinetic processes (Ren et al., 2020), including the first-pass effect and phase II conjugation. However, the metabolic detoxification pathways of amygdalin have not been well-clarified. Furthermore, there have also been no reports describing how Mahuang could influence the cyanogenic effects induced by amygdalin.

Ultrahigh-performance liquid chromatography coupled with high-resolution mass spectrometry (UPLC-HRMS) is a sensitive and potentially more robust technique than nuclear magnetic resonance and gas chromatography–mass spectrometry (GC-MS) for the identification of metabolites present in low concentrations and has better capacity to control the false discovery rate. A reliable and stable LC-MS/MS method is often used for the quantification of drugs and their metabolites in different matrices (Amer et al., 2017; Attwa et al., 2018a). In this study, a precise, accurate, and fast UPLC–tandem mass spectrometry (UPLC-MS/MS) method was used to measure the concentration of amygdalin and prunasin in rat plasma after oral administration. UPLC-HRMS was used to identify the metabolites of amygdalin *in vitro* and *in vivo*. We provide a comparative analysis of the stereoselective pharmacokinetics of amygdalin of the traditional decoction and dispensing granule formulation to clarify MX compatibility. Identification of metabolites and possible detoxification pathways of amygdalin *in vivo* was evaluated by incubating the preparation with rat liver microsomes and gut microbial enzymes. The possible effects of Mahuang on the detoxification pathway are also discussed later.

## Materials and Methods

### Chemicals and Materials

Amygdalin (99% purity, purchased from Sigma-Aldrich, Co.), prunasin (98% purity, purchased from Wuhan ChemFaces Biochemical Co., Ltd.), and benzaldehyde (98.5% purity, purchased from Shanghai Macklin Biochemical Co., Ltd.) were used as reference chemicals in the pharmacokinetics study. Diazepam (98% purity) and geniposide (97.5% purity), provided by the National Institutes for Food and Drug Control (Beijing, China), were selected as internal standards (IS) for studies of amygdalin pharmacokinetics and metabolism, respectively. Xingren (Gansu Province, batch number: 20170901) and Mahuang (Inner Mongolia Autonomous Region, batch number: 20180901) were purchased from Zisun Medicinal Material Co., Ltd (Guangzhou, China) and were authenticated by Professor Qunlin Zhang (School of Pharmacy, Anhui Medical University). The dispensing granules of Mahuang (1 g, equivalent to 5 g of the crude drug, batch number: 20170901) and Xingren (1 g, equivalent to 10 g of the crude drug, batch number: 20170901) were obtained from E-FONG Pharmaceutical Co. (Guangzhou, China). The inspection report of dispensing granules was provided by the E-FONG Pharmaceutical Co. (Supplementary Figure S1). Acetonitrile, methanol, and formic acid (HPLC grade) were purchased from Tedia Company, Inc (Fairfield, OH, United States). Ammonium acetate (HPLC grade) and SPE cartridges (ProElut, PLS 30 mg/ml) were provided by Dikma Technologies Inc (Lake Forest, CA, United States). Ultrapure water (18.2 MΩ) was purified by the Millipore system (Millipore Corp, Billerica, MA, United States). Ultrasonic cell disruption (JY92-II) was purified using a Scientz homogenizer. All reagents were analytical grades, unless otherwise stated. The BCA Protein Assay Kit for protein concentration quantification was purchased from Biosharp (Hefei, Anhui, China).

### Preparation of Herb Extracts

According to an extraction method described in TCM, Mahuang (90 g) and Xingren (90 g) were soaked in a moderate amount of water for 30 min and then extracted by boiling in 10-fold volumes of water (1800 ml) for 60 min. After filtering with absorbent gauze, an 8-fold volume of water was subsequently added to the residues and boiled for 90 min. The solution was filtered with five-layer absorbent gauze. The two filtrates were combined and concentrated to obtain the MX decoction. The obtained solution was concentrated to 180 ml (equal to 1 g crude herb/ml) using a rotary evaporator. The Xingren decoction was prepared following the same procedure. Finally, a 180 ml solution (equal to 0.5 g crude herb/ml) of Xingren (90 g) was obtained. The dispensing granule solution was prepared by dissolving the formula granules directly in water. Mahuang and Xingren were mixed in a 1:1 ratio (w/w, crude drug). We determined the concentrations of two main compounds (*D*-amygdalin and neoamygdalin) present in the Xingren decoction, MX decoction, Xingren granules, and MX granules (Supplementary Figure S2), the contents of which are shown in Supplementary Table S1, and other related components were characterized in a follow-up study.

### UPLC-MS/MS Conditions for Quantification

LC-MS/MS analysis was performed using a Shimadzu UPLC system (Shimadzu, Kyoto, Japan) coupled with a Triple Quada™ 5500 equipped with an electrospray ionization source (AB, Milwaukee, Wisconsin, United States). The UPLC system includes a DGU-20A5R online vacuum degasser, an LC-30AD pump, a SIL-30AC automatic sampler, a CBM-20A system controller, and a CTO-20AC column oven. Separation was carried out in a CDshell-RSP column (3.0 × 150 mm, 2.7 μm; Arlington, TX, United States), a novel chiral stationary phase composed of hydroxypropylated-β-cyclodextrin. The mobile phase at a flow rate of 0.4 ml/min consisted of phases A (5 mM ammonium acetate solution containing 0.1% formic acid) and B (methanol). Gradient separation was carried out by phase B of 12% during the first 2.5 min and then increasing from 12 to 95% during the following 4 min. Subsequently, phase B was linearly reduced to the initial ratio from 6.5 to 8 min. The temperatures of the column and the autosampler were fixed at 35°C and 4°C, respectively. A 5 µl sample was loaded into the system.

Tandem MS was equipped with an electrospray ionization interface operating in the positive ion mode. For the optimization of each multiple reaction monitoring (MRM) parameter, other normal LC-MS/MS conditions were used, including ion source parameters (spray voltage: 5500 V; atomization temperature: 500°C; nebulizer pressure: 35 psi). The optimized quantitative ion pair (m/z) and qualitative ion pair selected for the MRM mode used the following optimized ion transitions for each precursor/product pair: amygdalin (457.3→163.3, 90 V, 22 eV), prunasin (313.0→163.1, 90 V, 14 eV), and diazepam (285.2→228.0, 140 V, 30 eV). AB-SCIEX Analyst (version 1.6.3) software was used for data acquisition and processing.

### Preparation of Standard and Quality Control Samples

Standard solutions were obtained by mixing and serial dilution of stock solutions of amygdalin (914.8 μg/ml) and prunasin (1,181.4 μg/ml). The diazepam (IS, 10.0 ng/ml) was prepared by dilution of its stock solution (1.0 mg/ml). Acidic conditions are required to prevent its epimerization. Next, stock solutions and further dilutions were prepared in 50 and 10% aqueous methanol solution (v/v, containing 0.1% formic acid), respectively. Calibration curves and quality control (QC) samples were prepared by spiking 5 μl of working standard with 95 μl blank rat plasma. The calibration curve of amygdalin and prunasin was prepared in the concentration range between 1.0 and 128.1 ng/ml and 4.6 and 590.7 ng/ml, respectively. Three levels of QC samples were prepared containing amygdalin (2.2, 22.0, and 109.8 ng/ml) and prunasin (9.13, 91.3, and 456.5 ng/ml). All solutions were stored at 4°C and brought to room temperature prior to use.

### Preparation of Rat Plasma Samples

For the pharmacokinetics study, 100 μl rat plasma was transferred into a 1.5-ml polypropylene centrifuge tube, followed by the addition of 400 μl of diazepam (IS, 10.0 ng/ml). The samples were mixed and loaded into an SPE cartridge which was preconditioned with methanol and deionized water. The cartridge was washed with 1 ml of 10% aqueous methanol solution containing 0.1% formic acid and vacuum-dried for 1 min. The analytes were then eluted with 1 ml of methanol containing 0.1% formic acid. The eluent was evaporated to dryness under a gentle stream of nitrogen gas. The residue was resuspended with 400 µl of 5 mM ammonium acetate containing 0.1% formic acid and centrifuged (15,000×g for 8 min) for quantitative analysis.

### Analytical Method Validation

The method was validated for specificity, linearity, sensitivity, accuracy and precision, matrix effect, extraction recovery, and stability of the analytes in rat plasma (Commission, 2015).

### Pharmacokinetics of Amygdalin in Traditional Decoction and Dispensing Granule

Male Sprague–Dawley rats, of specific-pathogen-free grade, were obtained from the Anhui Medical University Laboratory Animal Center (Certificate number SCXK 2017–001). All animal experiments were approved by the Experimental Animal Ethics Committee of Anhui Medical University (LLSC20170348) and complied with the requirements of the Animals Ethics Procedures and Guidelines of the People’s Republic of China. They were housed under controlled environmental conditions (ambient temperatures 23–25°C; 12-h light/12-h dark cycles, 45–55% relative humidity) and fed ad libitum for the first 2 weeks. Rats were fasted for 12 h with free access to water prior to and during the experiment.

The same dose of crude extract (3 g/kg) was administered orally to rats in these four groups, including the Xingren decoction (equivalent to 44.1 mg/kg *D-*amygdalin and 40.2 mg/kg neoamygdalin), Xingren dispensing granules (equivalent to 45.3 mg/kg *D*-amygdalin and 50.7 mg/kg neoamygdalin), MX decoction (equivalent to 38.4 mg/kg *D*-amygdalin and 14.5 mg/kg neoamygdalin), and MX dispensing granules (equivalent to 24.3 mg/kg *D*-amygdalin and 25.7 mg/kg neoamygdalin). A 500 μl sample of orbital venous blood was collected in heparinized 1.5-ml polythene tubes before the treatment and subsequently at 5 min, 0.25, 0.5, 0.75, 1, 1.5, 2, 3, 4, 5, 6, 9, and 12 h after dosing. Plasma samples were separated immediately from blood by centrifugation at 4,500×g for 10 min and stored at −80°C until analysis.

### Extraction of Gut Microbial Enzyme

Rat fecal specimens (approximately 0.5 g each) were dissolved in 10-fold PBS (m/v) and centrifuged (3,500×g for 15 min at 4°C) to remove the supernatant. The precipitate was washed twice and resuspended in cold PBS mentioned earlier. The resulting suspension was sonicated (300W, 10 s over 20 s intervals) for 45 min in an ice bath to rupture bacterial cells and then centrifuged at 13,000×g for 30 min at 4°C to obtain a suspension containing microbial enzymes and to remove debris (Sun et al., 2020), and aliquots of the supernatant were subsequently stored in a refrigerator until use.

### UPLC-HRMS Conditions for Metabolite Separation and Detection

A UHPLC Dionex Ultimate 3,000 (Thermo Scientific, San Jose, United States) equipped with a cooling autosampler and column oven was utilized. Separation was carried out on an HSS T3 column (2.1 × 100 mm, 1.8 µm, Waters, Ireland) with a column temperature maintained at 45°C. Following the gradient elution program with the same mobile phase composition was established: 0–3 min, 5% B; 3–5 min, 5–25% B; 5–11 min, 25% B; 11–13.8 min, 60% B; 13.9–16 min, 5% B. The injection volume was 2 µl, and the flow rate was 0.25 ml/min.

A Q-Exactive plus hybrid quadrupole-orbitrap mass spectrometer (Thermo Scientific, San Jose, United States) with a heat electrospray ionization (HESI) probe was employed using a data-dependent MS/MS approach in a negative or positive ion mode. The mass conditions were optimized as follows: capillary temperature, 320°C; spray voltage, −3.5 kV; S-lens RF level, 50 V; auxiliary gas heater temperature, 200°C; *s*heath gas pressure, 40 psi; *a*uxiliary gas pressure, 10 psi; scan range, 100–1,000 m/z; resolution, 17,500; solation window: 2.0 m/z; stepped normalized collision energy: 20, 40, 60; dynamic exclusion, 10 s; and apex trigger, 2–6 s. Nitrogen was used for spray stabilization, for high-energy collision dissociation, and as the damping gas in the C-trap. Q-Exactive 2.9, Xcalibur 4.1, Compound Discoverer 3.0, and MS Frontier 7.0 software (Thermo Fisher Scientific, San Jose, United States) were used for instrument control, data acquisition, and analysis.

### Metabolic Transformations of Amygdalin in the Traditional Decoction and on Exposure to Rat Liver Microsomes and Gut Microbial Enzymes

In the TCM theory, the proper combination of herbs is considered essential to enhance therapeutic effects and reduce toxic side effects. Herb–herb interactions, inducing changes in the solubility or generation of a new compound, may occur during the decoction process. Liver and gut microbes play an important role in drug metabolism (Knudsen et al., 2021; Sodhi and Benet, 2021). Thus, chemical transformations or endogenous metabolism of amygdalin was investigated during decoction and incubation of the rat liver microsome (RLM) and gut microbial enzyme (RGME) preparations, respectively.

In this study, RLM was prepared according to the hypothermal differential centrifugation method, as described in the literature (Cheng et al., 2019). The liver tissue was homogenized with four volumes (w/v) of ice-cold PBS buffer. The homogenate was centrifuged at 9,000 g for 20 min at 4°C. Then the supernatant was centrifuged at 100,000×g for 60 min at 4°C, and the pink precipitate was used as the liver microsome fraction. The protein concentration of RLM and RGME was estimated using the BCA and subsequently aliquoted and stored in a refrigerator until use. The incubation solution for RLM consisted of RLM (0.5 mg/ml), 50 mM PBS (pH = 7.4), and the NADPH regeneration system (containing 1.5 mM NADP ^+^, 5 mM glucose-6-phosphate, 5 mM MgCl_2_, and 1 unit/ml G6PDH). The reaction was started by adding 10 μl NADPH. The incubation preparation for RGME consisted of PBS, D-amygdalin, and RGME and was carried out at 37°C for 2 h, and the reaction was terminated by adding 3-fold ice-cold acetonitrile. Furthermore, 20 μM (42 μg/ml) D-amygdalin was added to an already prewarmed incubation system (800 μl).

All samples, including the incubation mixture of the traditional decoction and the dispensing granule solution, were filtered using a 0.22-µm nylon filter after a tenfold dilution with water, after which the samples were purified with the SPE protocol, as described before for the preparation *rat plasma samples*. The supernatant was obtained by centrifugation at 16,000×g for 10 min, and the residues were dried under gentle nitrogen steam. The residues were suspended with 400 μl initial mobile phase and then filtered through a syringe with a 0.22-µm nylon membrane. Principal component analysis (PCA) (Peterson et al., 2016) was performed to evaluate the changes in metabolite composition associated with amygdalin at a normalized concentration (40 μM, 84 μg/ml) of the decoction and dispensing granules.

### Data Handing and Statistical Analysis

Pharmacokinetic parameters including half-life (t_1/2_), maximum plasma concentration (C_max_), the time for maximum concentration (T_max_), the area under the curve (AUC_0–t_), the volume of distribution (Vz), and the clearance rate (Hulin et al., 2019) were estimated by noncompartmental analysis using Phoenix WinNonlin software (version 8.3.1.5014, Pharsight, United States).

Generally, metabolites with the same or similar parent nucleus always have similar cleavage fragments in the ESI-MS^n^ spectrum, so it is possible to identify metabolites and even the corresponding metabolic pathways. First, the MS^1^ (primary level of MS) and MS^2^ (secondary level of MS) spectrum data of amygdalin were collected to determine its MS/MS fragment behavior (precursor–product ion relationships). Next, Compound Discoverer 3.0 was employed to analyze the metabolites of amygdalin and generated extracted ion chromatograms (XICs) with retention time, accurate mass, and isotopic pattern. Considering a significant mass intensity, a suitable tolerance window for XIC extraction (±5 ppm), and established fragment ions, the structure of possible metabolites of amygdalin could be identified. Finally, the fragmentation behavior analysis predicted by Mass Frontier software was combined with the analysis of known metabolic pathways to assess whether the identification of metabolites was reasonable or not.

Variations in the chemical profile of amygdalin between the traditional decoction and dispensing granules at a normalized concentration of Xingren were explored by principal component analysis (PCA). All data are presented as mean ± SD. Statistical analysis was performed using the single-factor ANOVA. A *p* value less than 0.05 was considered statistically significant. Statistical analysis was performed using SPSS v.20 (IBM Corp., Armonk, NY, United States).

## Results

### Method Validation

The validated method showed good sensitivity, selectivity, and reproducibility. The XICs of blank rat plasma, blank plasma spiked with IS and analytes at LLOQ, and plasma samples after administration of traditional decoction and dispensing granules are shown in Supplementary Figure S3. No obvious interference was found in the retention time. Eight-point calibration plots exhibited satisfactory linearity (correlation coefficient >0.99) in the concentration range of 1.00–128.1 and 4.62–590.7 ng/ml for amygdalin and prunasin (Supplementary Table S2). Reproducible extraction recovery (79.6–90.3%, CV ≤ 7.01%) and the neglectable matrix effect (112.6–114.7%, CV ≤ 8.74%) were observed (Table 1). Acceptable intraday and intraday precision and accuracy were demonstrated (88.3–113.0%; CV ≤ 13.3%) for matrix QC samples (Supplementary Table S3). In addition, both amygdalin and prunasin were quite stable under current storage and processing conditions, with variability ranging from −5.7 to 14.7% (CV ≤ 14.3%) (Supplementary Table S4).

**TABLE 1:** Recovery and matrix effect of amygdalin and prunasin in rat plasma using solid-phase extraction (*n* = 6).

Analyte	Concentration (ng·mL^−1^)	Recovery (%)		Matrix effect (%)	
		Mean ± SD	CV (%)	Mean ± SD	CV (%)
Amygdalin	2.20	79.6 ± 0.93	1.17	112.6 ± 9.83	8.74
	22.0	82.5 ± 4.34	5.26	113.1 ± 8.24	7.29
	109.8	85.9 ± 4.03	4.70	113.1 ± 5.51	4.87
Prunasin	9.13	90.3 ± 3.54	3.91	114.7 ± 3.52	3.07
	91.3	85.4 ± 5.21	6.10	113.9 ± 8.84	7.76
	456.5	87.4 ± 6.13	7.01	113.2 ± 3.90	3.44

CV: coefficient of variation.

### Bioequivalence of Dispensing Granule Was not Consistent With the Traditional Decoction

**FIGURE 1:**
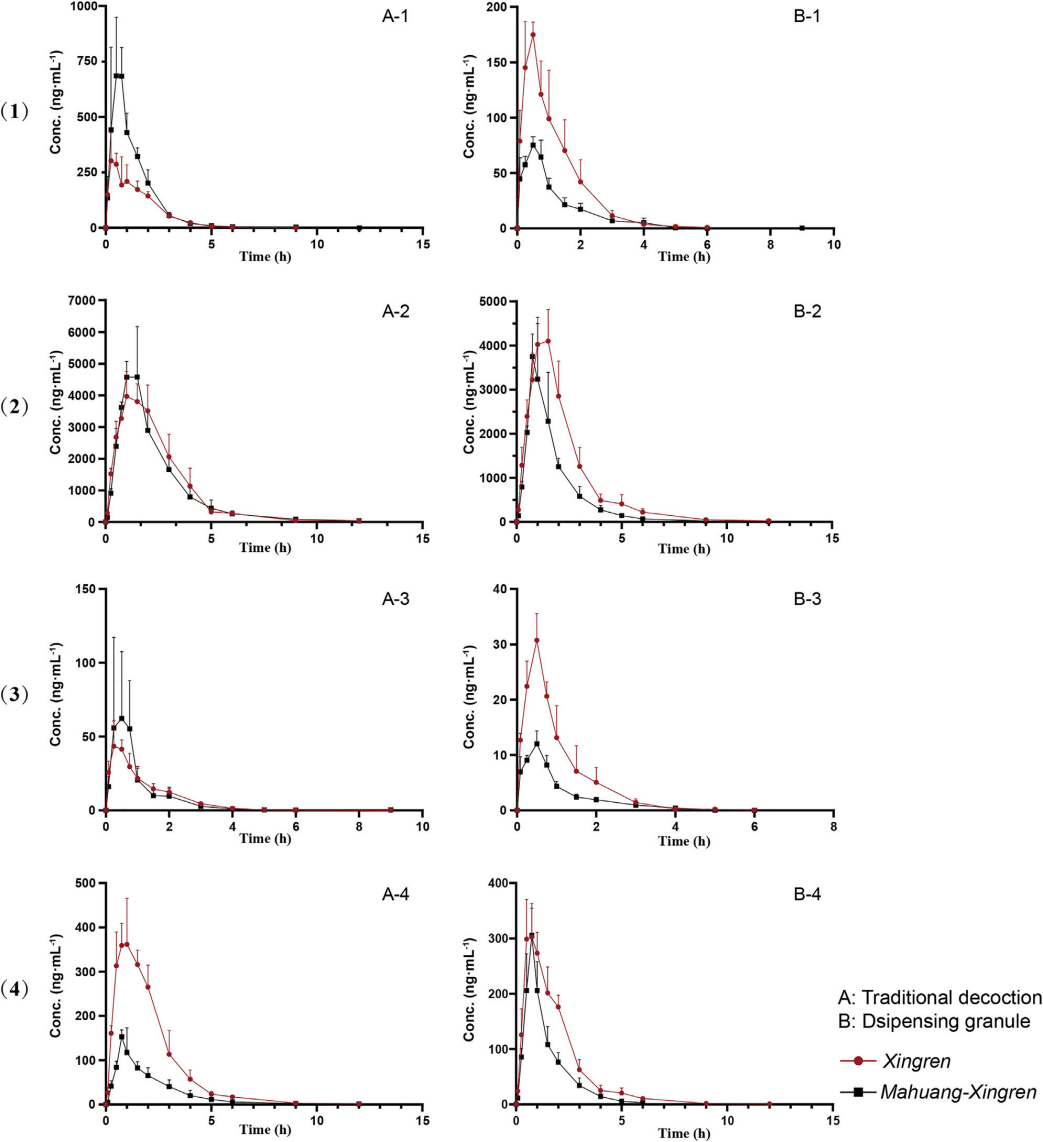
Mean plasma concentration profiles over time for **(1)** *D*-amygdalin, **(2)** neoamygdalin, **(3)** *D*-prunasin, and **(4)** sambunigrin in rat (mean ± SD, *n* = 5) following oral administration of Xingren, Mahuang–Xingren decoction, and dispensing granule preparations.

The mean plasma concentration–time profiles (*n* = 5) are shown in Figure 1. Plasma concentration versus time was simulated and followed the first-order rate law. Therefore, dose-normalized C_max_ and AUC_0-t_ (C_max_/D and AUC_0-t_/D) were used to assess drug exposure based on the different concentrations of amygdalin in the decoction or the dispensing granule. Prunasin, presenting a cleaved glucose molecule, was the primary deglycosylated metabolite of amygdalin. Both amygdalin (including *D*-amygdalin and neoamygdalin) and prunasin (including *D*-prunasin and sambunigrin) (Supplementary Figure S2) were used to estimate the pharmacokinetic properties in the following analysis.

No significant differences in T_max_ or t_1/2_ of amygdalin and prunasin (except the shorter T_max_ of prunasin in the dispensing granule preparation) were observed in the MX combination. However, the C_max_/D and AUC_0-t_/D of amygdalin (except for the dispensing granule) and prunasin were all improved with the addition of Mahuang (*p <* 0.05 or *p <* 0.01) (Tables 2, 3). Higher plasma exposure (presented as C_max/_and AUC) of amygdalin and prunasin was achieved in the MX decoction (*p <* 0.05 or *p <* 0.01). In addition, the clearance (*p <* 0.01) and the biodistribution of amygdalin (*p <* 0.05) were markedly lower than for those of the dispensing granule preparation (Supplementary Tables S5, S6). In summary, Mahuang increased the bioavailability of total amygdalin, which was consistent with our previous report. Meanwhile, reduced drug clearance and biodistribution were only confirmed for the decoction. The data suggested that the traditional decoction was superior in terms of its lower toxicity and also showed better therapeutic efficacy than the MX dispensing granule preparation.

**TABLE 2:** Pharmacokinetic parameters of *D*-amygdalin, neoamygdalin, *D*-prunasin, and sambunigrin in rats after oral administration of Xingren and MX decoction.

Compound	Group	AUC_0-t_/dose (h·ng·mL^−1^)	C_max_/dose (ng·mL^−1^)	T_max_ (h)	t_1/2_ (h)	CL (L·h^−1^·kg^−1^)	V_d_ (L·kg^−1^)
*D*-amygdalin	Xingren	11.46 ± 2.1^#;#^	6.61 ± 2.12^#;#^	0.38 ± 0.14	0.85 ± 0.10	89.24 ± 17.16^##^	109.59 ± 23.33^##^
	MX	31.39 ± 4.60**^&&^	24.11 ± 4.71**^&&^	0.50 ± 0.20	1.39 ± 0.92	32.19 ± 4.09**^&^	66.34 ± 48.44^&^
Neoamygdalin	Xingren	1.56 ± 0.32	1.18 ± 0.38	0.38 ± 0.14	0.97 ± 0.25	660.25 ± 142.11	920.43 ± 266.32
	MX	5.77 ± 3.43*	6.22 ± 4.39*	0.50 ± 0.20	0.97 ± 0.33	224.84 ± 134.24*	315.35 ± 121.87*
*D*-prunasin	Xingren	247.91 ± 40.24^##^	86.61 ± 15.50^##^	1.25 ± 0.50	1.50 ± 0.23	4.10 ± 0.77^##^	8.95 ± 2.73^##^
	MX	355.39 ± 50.39*^&&^	163.11 ± 32.69*^&&^	1.13 ± 0.25	1.82 ± 0.58	2.83 ± 0.44*^&&^	7.25 ± 1.71^&&^
Sambunigrin	Xingren	23.80 ± 2.08	10.50 ± 1.18	0.81 ± 0.24	1.33 ± 0.25	42.13 ± 3.98	81.32 ± 22.14
	MX	24.53 ± 6.63	13.94 ± 2.0*	0.81 ± 0.13	1.12 ± 0.34	42.53 ± 10.32	65.62 ± 8.45

*p < 0.05, **p < 0.01 for Xingren*. vs*. MX (Mahuang–Xingren).

^#^ *p* < 0.05., ^##^ *p* < 0.01 for neoamygdalin *vs. D-*amygdalin *and* sambunigrin *vs. D-*prunasin in Xingren group.

^&^ *p* < 0.05, ^&&^ *p* < 0.01 for neoamygdalin *vs. D-*amygdalin and sambunigrin *vs. D-*prunasin in the MX group.

**TABLE 3:** Pharmacokinetic parameters of *D*-amygdalin, neoamygdalin, *D*-prunasin, and sambunigrin in rats after oral administration of Xingren and MX dispensing granules.

Compound	Group	AUC_0-t_/Dose (h·ng·mL^−1^)	C_max_/Dose (ng·mL^−1^)	T_max_ (h)	t_1/2_ (h)	CL (L·h^−1^·kg^−1^)	V_d_ (L·kg^−1^)
*D*-amygdalin	Xingren	4.94 ± 1.24^##^	3.74 ± 0.28^##^	0.44 ± 0.13	0.84 ± 0.19	214.21 ± 57.76^##^	262.51 ± 117.45^#^
	MX	4.69 ± 0.62^&&^	3.63 ± 0.33^&&^	0.46 ± 0.28	0.59 ± 0.27	215.65 ± 30.62^&&^	182.08 ± 84.12^&&^
Neoamygdalin	Xingren	0.78 ± 0.20	0.72 ± 0.11	0.50 ± 0.00	0.85 ± 0.34	1,355.38 ± 422.24	1,649.78 ± 762.94
	MX	0.70 ± 0.08	0.64 ± 0.10	0.46 ± 0.28	0.62 ± 0.27	1,438.09 ± 146.74*	1,312.94 ± 679.68
*D*-prunasin	Xingren	213.71 ± 23.68^##^	95.74 ± 7.24^##^	1.25 ± 0.29^#^	1.56 ± 0.18	4.70 ± 0.57^##^	10.53 ± 1.57^##^
	MX	278.90 ± 33.00*^&&^	183.14 ± 36.83**^&&^	0.81 ± 0.13*	1.74 ± 0.55	3.61 ± 0.45*^&&^	8.83 ± 1.86^&&^
Sambunigrin	Xingren	15.23 ± 1.28	8.03 ± 0.94	0.69 ± 0.13	1.29 ± 0.27	65.81 ± 5.71	121.44 ± 24.13
	MX	20.15 ± 2.57*	15.78 ± 2.51**	0.75 ± 0.00	0.88 ± 0.09*	49.70 ± 5.83**	63.42 ± 12.15**

*p < 0.05, **p < 0.01 for Xingren*. vs*. MX (Mahuang–Xingren).

^#^ *p* < 0.05, ^##^ *p* < 0.01 for neoamygdalin *vs. D-*amygdalin and sambunigrin *vs. D-*prunasin in the Xingren group.

^&^ *p* < 0.05, ^&&^ *p* < 0.01 for neoamygdalin *vs. D-*amygdalin and sambunigrin *vs. D-*prunasin in the MX group.

*D*-amygdalin appears to be better absorbed than neoamygdalin due to its higher C_max_/D and AUC_0-t_/D (Supplementary Table S7). Previous studies have also indicated that antagonism of *D*-amygdalin (i.e., amygdalin) metabolism may be critical for detoxification in the MX combination. The ratio of *D*-amygdalin to neoamygdalin was considered an exposure index of *D*-amygdalin *in vivo*. The variations of this ratio were compared for the MX combination and the single herbs. As shown in Supplementary Table S7, the ratio of AUC_0-t_/D in the MX decoction decreased from 7.19 to 5.72 (*p <* 0.05) (by approximately 20.4%). On the contrary, no significant decrease was observed for the dispensing granule preparations. These findings indicated that Mahuang decreased the risk of exposure relative to *D*-amygdalin and reduced its toxicity compared to traditional decoction. In general, better bioavailability of *D*-amygdalin, neoamygdalin, *D*-prunasin, or sambunigrin in the decoction was observed than the dispensing granules, which indicated that *in vivo* drug disposition of *D*-amygdalin differed significantly for the two combinations (Supplementary Tables S5, S6). MX decoctions should not be completely replaced by the dispensing granule formulation in terms of toxicity.

### Identification of Metabolites and the Metabolic Detoxification Pathway of Amygdalin

By comparing the retention time, exact mass and possible MS/MS fracture behavior of metabolites, a total of 16 different compounds were identified or tentatively characterized (Table 4). The fragmentation patterns of the 16 compounds are shown in Supplementary Figure S4. The retention time of amygdalin amide was 7.14/7.24 min, and the MS spectrum showed [M + HCOO]^-^ ion at m/z 520.16608 (C_20_H_27_NO_11_, 0.58 ppm). According to the representative product ion (m/z 474.16170, 323.09837, 179.05611), it could be inferred that the nitrile group was introduced into H_2_O. Therefore, it was presumed to be the amidated product of amygdalin epimers.

**TABLE 4:** Identification of amygdalin-related metabolites in gut microbial enzyme incubation system by HR-MS.

Peak no.	t_R_	Molecular formula	Measured mass (Da)	Theoretical mass (Da)	Error (ppm)	MS/MS	Identification
1	9.19	C_20_H_27_NO_11_	502.15576	502.15552 [M + HCOO]^-^	0.43	456.15114, 323.09837, 179.05611	Amygdalin
2	9.19	C_21_H_29_NO_11_	516.17102	516.17116 [M + HCOO]^-^	0.05	456.15114, 323.09837	Methyl amygdalin
3	10.33/10.62	C_14_H_17_NO_6_	340.10333	340.10269 [M + HCOO]^-^	1.88	294.09831,188.05645, 161.04555	Prunasin
4	10.33/10.62	C_15_H_19_NO_6_	354.11945	354.11834 [M + HCOO]^-^	3.13	188.05645, 161.04555	Methyl prunasin
5	2.32	C_14_H_15_NO_7_	354.08160	354.08196 [M + HCOO]^-^	0.98	308.07758, 290.06701, 175.02481, 131.03498	Laetrile
6	8.69	C_8_H_7_NO	178.05020	178.04987 [M + HCOO]^-^	1.86	178.05097,132.04549	Mandelonitrile
7	14.12	C_9_H_9_NO	148.07552	148.07569 [M + H]^+^	1.20	148.07569, 130.06513	Methyl mandelonitrile
8	14.25	C_7_H_6_O	151.03882	151.03897 [M + HCOO]^-^	1.01	151.04007, 123.04515	Benzaldehyde
9	7.14/7.24	C_20_H_29_NO_12_	520.16638	520.16608 [M + HCOO]^-^	0.58	474.16170, 323.09837, 312.10888, 179.05611,150.05605	Amygdalin amide
10	9.17	C_20_H_28_O_13_	521.15118	521.15009 [M + HCOO]^-^	2.08	475.14572, 431.15589, 269.10306, 161.04555	Amygdalin acid
11	8.84	C_20_H_30_O_12_	507.17139	507.17083 [M + HCOO]^-^	1.09	461.16645, 299.11363	M1: HPTTHME
12	6.77	C_14_H_19_NO_7_	358.11404	358.11326 [M + HCOO]^-^	2.20	312.10888, 161.04555, 150.05605, 101.02442	Prunasin amide
13	7.22/7.50	C_14_H_18_O_8_	313.09271	313.09179 [M-H]^-^	2.93	313.09289, 151.04007	Prunasin acid
14	1.17	C_8_H_9_NO_2_	169.09694	169.09715 [M + NH_4_]^+^	1.27	169.09715, 152.07061, 134.06004	Mandelonitrile amide
15	9.44	C_8_H_10_O_2_	156.10191	156.10190 [M + NH_4_]^+^	0.05	139.07536, 97.06479	M2: HPE
16	14.18	C_8_H_10_O	140.10692	140.10699 [M + NH_4_]^+^	0.52	140.10699, 122.09643	M3: PE

M1: 2- hydroxyphenyl-2-[3,4,5-trihydroxy-6-(3,4,5-trihydroxy-6-hydroxymethyl oxan-2-yloxy)-methyl oxan-2-yloxy] ethane (HPTTHME).

M2: 2-hydroxy-2-hydroxyphenyl ethane (HPE).

M3: 2-hydroxy-2-phenyl ethane (PE).

**FIGURE 2:**
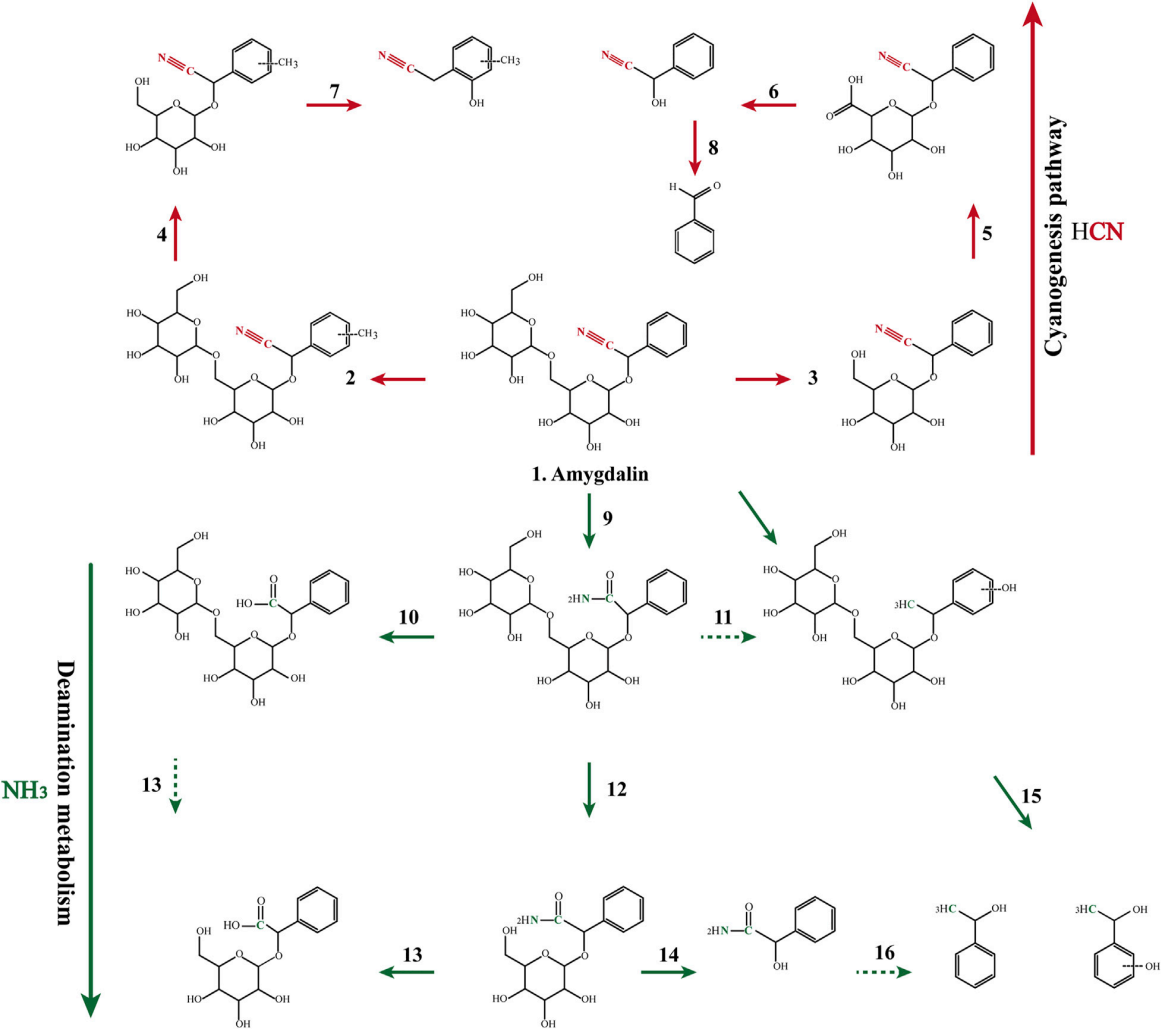
Proposed major metabolic pathways for amygdalin occurring in the incubation system and in the decoction preparation.

The drug incubation test indicated that amygdalin can be deglycosylated by β-glucosidase to yield prunasin, mandelonitrile, benzaldehyde, and laetrile, which is recognized as a classical metabolism pathway in many studies. Furthermore, amidated, carboxylic, hydroxylated, and methylated metabolites were also identified (Figure 2; Table 4). The *in vitro* half-life (t_1/2_) indicated that the metabolism of amygdalin in RGME was superior to that obtained in the RLM preparation (Supplementary Table S8). Similarly, the CL_int_ obtained *in vitro* indicated that amygdalin achieved a higher clearance rate in RGME than in RLM, which also demonstrated that the gut microbiota may perform the primary functions in amygdalin metabolism.

**FIGURE 3:**
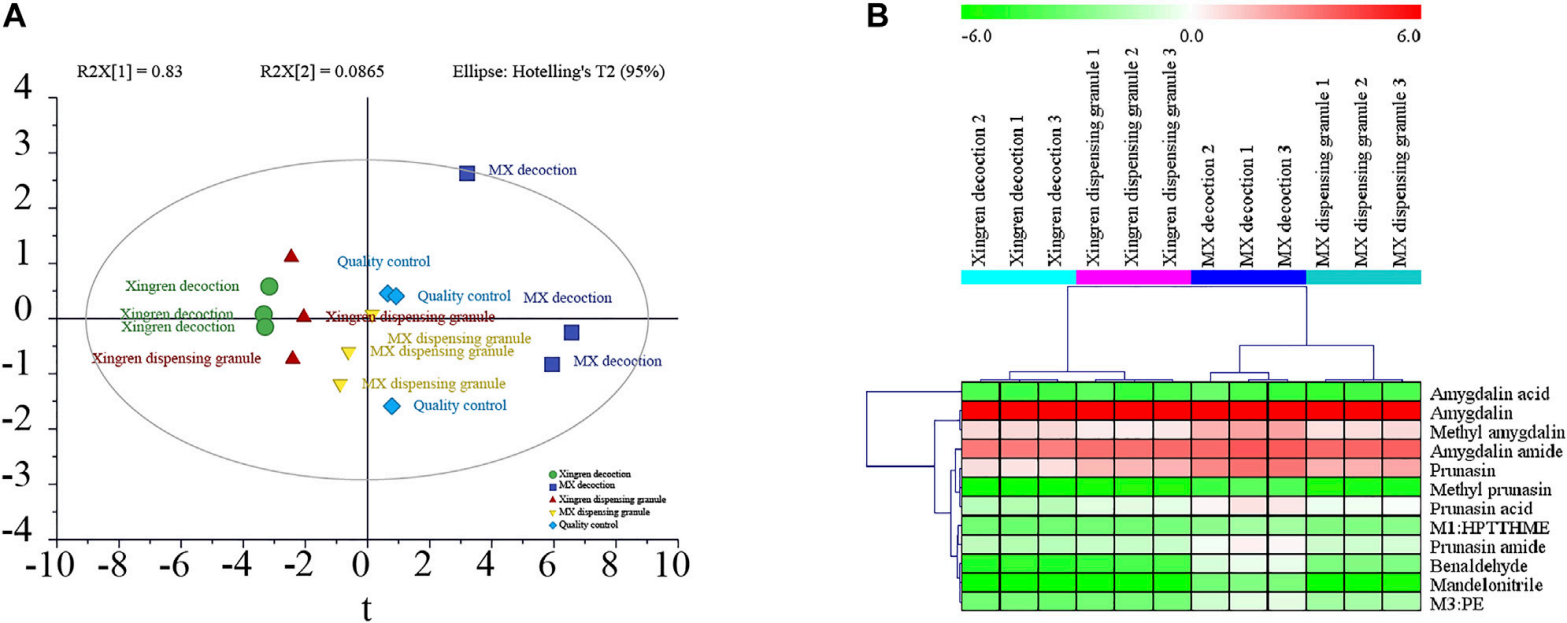
The PCA plots **(A)** and heatmap **(B)** of 16 compounds in Xingren decoction, MX (Mahuang–Xingren) decoction, Xingren dispensing granules, and MX dispensing granules.

As shown in Figure 2, the cyanogenesis and non-cyanogenesis metabolism pathways depended on whether the carbon and nitrogen atoms presented a triple bond. Amination and carboxylation processes were demonstrated to be the detoxification pathways of amygdalin-induced cyanide toxicity. Compounds derived from the traditional decoction and dispensing granules were identified or tentatively characterized according to the 16 compounds emerging from the MS/MS analysis (Supplementary Figure S5). The chemical transformation of amygdalin was compared for the traditional decoction and dispensing granule preparations. As shown in Figure 3, obvious metabolic transformations occurred in the MX decoction preparation compared to the dispensing granule preparation. Overall, these results indicated that the detoxification of Xingren might occur during the boiling of the preparation of the herbal mixture and by metabolism *in vivo*.

## Discussion

### Optimization of UPLC-MS/MS Condition

Amygdalin and prunasin, glycosides with high polarity and inefficient protonation, showed weak signal intensity in the MS/MS electrospray ionization mode. Although the bioanalysis of amygdalin has been reported in several pharmacokinetic studies, satisfactory chiral separation has not been achieved of the two epimers to date (Picmanova et al., 2015). Herein, LC-MS/MS optimization was performed by selecting optimal mobile-phase modifiers, to achieve the chiral separation of the epimers, and by adjusting the SPE conditions in the sample preparation.

The addition of a hydrogen cation [M + H]^+^ or of a sodium ion [M + Na]^+^ was observed in precursor ion scans, but no significant or stable product ions were detected with increasing collision energy. The adduct ion of ammonium [M + NH_4_]^+^ was shown to achieve high abundance and reproducible precursor and product ions. Cyclodextrin-based chiral stationary phases were also obtained successfully for the chromatography separation of chiral molecules with aromatic substituents (Li et al., 2017). The hydroxylpropyl group of the derivatized cyclodextrins extended beyond the mouth of the cyclodextrin cavity. Considering the superiority of liquid chromatographic enantiomer separation, CDShell-RSP (beta-cyclodextrin, hydroxylpropyl), the best column stationary phases available for superficially porous particle silica, was adopted to separate the two epimers of amygdalin and prunasin (Barhate et al., 2017). As shown in Supplementary Figure S3, the retention times of *D-*amygdalin, neoamygdalin, *D*-prunasin, and sambunigrin were 3.52, 3.20, 4.22, and 5.17 min, respectively, with similar baseline resolution. Protein precipitation and liquid–liquid extraction were performed according to published data, but the epimers could not be separated efficiently due to a higher proportion of organic solvents. Nitrogen steam evaporation has also been shown to be time-consuming (Zhao et al., 2021b). SPE, or conditioning, loading of the sample, washing, and elution (CLWE), is an efficient protocol for sample preparation in LC-MS/MS quantification (Bury et al., 2018). In this study, the composition of the washing solvent was optimized for reproducible recovery and acceptable matrix interference using gradient elution. The data indicated that satisfactory extraction of amygdalin and prunasin was achieved using a 10% methanol aqueous solution containing 0.1% formic acid.

### MX Compatibility Superiority: Decoction Maintain a Balance Between Therapeutic Effectiveness and Safety

In our previous urinary excretion study, the XIC of MRM chromatograms of amygdalin displayed two pairs of peaks indicating different retention times, suggesting that there could still be an unknown metabolite that shares the same chemical structure as amygdalin. Amygdalin may be aminated, methylated, hydroxylated, and so on *in vivo* (Attwa et al., 2018b). In this study, a powerful LC-HRMS method was employed to screen and identify the potential metabolites of amygdalin. The deamination metabolism pathway was first proposed as the detoxification pathway based on the identification of amygdalin amide and other metabolites. Interestingly, the pathway was also identified during the preparation of the MX decoction. These results suggested that the traditional MX decoction was superior to its single-herb application in maintaining a balance between therapeutic effectiveness and safety. Conversely, MX reduced the relative exposure risk of *D*-amygdalin and increased the bioavailability of amygdalin. In contrast, amygdalin might be partially detoxified by MX decoction and by *in vivo* metabolism. Herb pairings embody the balanced wisdom of Chinese herbal compatibility (Wang et al., 2020), which is also confirmed by the MX compatibility of the stereoselectivity and deamination of amygdalin.

## Conclusion

Lower exposure of *D*-amygdalin and a higher abundance of detoxified metabolites were present in the MX decoction. Based on our findings, stereoselective pharmacokinetics and deamination metabolism are the detoxification pathways underlying the compatibility of the MX preparation. Thus, MX decoctions should not be completely replaced by dispensing granules from the perspective of toxicity. However, the detoxification mechanism of amygdalin was not well-presented in this study. The deamination pathway of amygdalin *in vivo* and its regulation by Mahuang remain unclear and merit further investigation. Therefore, transport mechanisms are being explored using the Caco-2 cell model.

## Data Availability

The datasets generated for this study are available on request to the corresponding authors.
