# Corrigendum: Interactions Between *Ephedra sinica* and *Prunus armeniaca*: From Stereoselectivity to Deamination as a Metabolic Detoxification Mechanism of Amygdalin

**DOI:** 10.3389/fphar.2021.831921

**Published:** 2022-01-05

**Authors:** Yan Qin, Shanshan Wang, Qiuyu Wen, Quan Xia, Sheng Wang, Guanjun Chen, Jiayin Sun, Chenlin Shen, Shuai Song

**Affiliations:** ^1^ Inflammation and Immune Mediated Diseases Laboratory of Anhui Province, Anhui Institute of Innovative Drugs, Institute for Liver Diseases of Anhui Medical University, School of Pharmacy, Anhui Medical University, Hefei, China; ^2^ Department of Pharmacy, The First Affiliated Hospital of Anhui Medical University, Hefei, China; ^3^ The Grade 3 Pharmaceutical Chemistry Laboratory of State Administration of Traditional Chinese Medicine, Hefei, China; ^4^ Center for Scientific Research of Anhui Medical University, Hefei, China; ^5^ Hefei Kaifan Analytical Technology Co., Ltd., Hefei, China

**Keywords:** amygdalin, LC-MS/MS, pharmacokinetic, stereoselectivity, compatibility, metabolism, detoxification

In the published article, there was an error in affiliations for “Shuai Song” and “Quan Xia.” They should not have affiliation 1. The correct affiliations are: “Shuai Song^2,3^, Quan Xia^2,3^.”

In the original article, there was an error. The concentration of amygdalin and prunasin in QC samples stated in **Preparation of Standard and Quality Control Samples** were reversed.

A correction has been made to **Preparation of Standard and Quality Control Samples**, as follows:

“Three levels of QC samples were prepared containing amygdalin (2.2, 22.0, and 109.8 ng/ml) and prunasin (9.13, 91.3, and 456.5 ng/ml).”

The authors apologize for this error and state that this does not change the scientific conclusions of the article in any way. The original article has been updated.

