# Supplementary material for: Interactions Between Ephedra sinica and Prunus armeniaca: From Stereoselectivity to Deamination as a Metabolic Detoxification Mechanism of Amygdalin
Source: Front Pharmacol. 2021 Nov 26;12:744624. doi: 10.3389/fphar.2021.744624 (PMC8661500; doi:10.3389/fphar.2021.744624)
Supplement: Supplementary file 1 [file DataSheet1.docx]

Supplementary Material

**
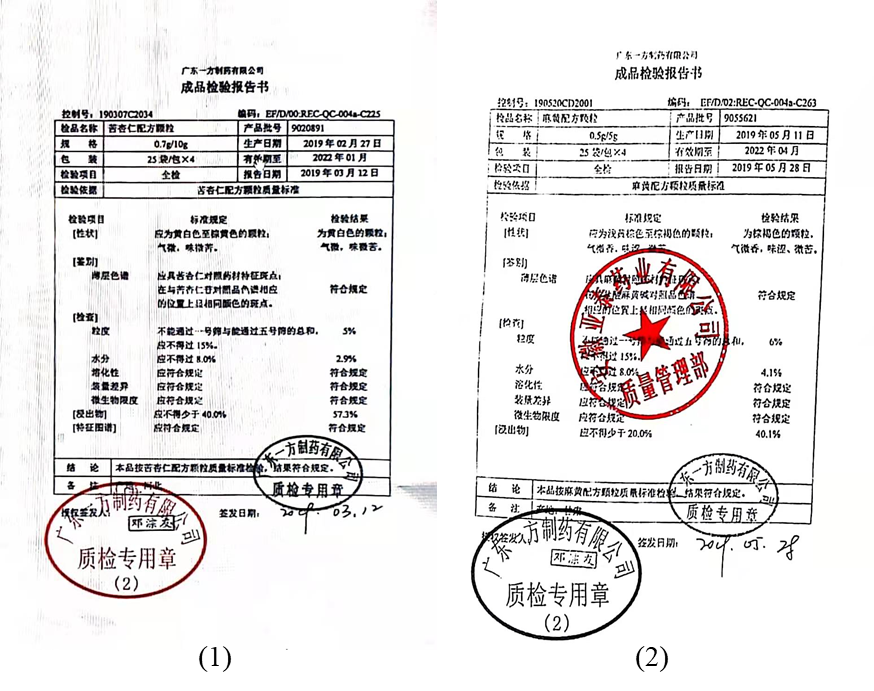
**

**Figure 1.** The factory inspection report of Xingren (1) and Mahuang-Xingren (2) dispensing granules.





**Figure 2.** Chemical structures of 2 active components (*D*-amygdalin and neoamygdalin) contained in Xingren decoction, MX decoction, Xingren granules, and MX granules and its metabolites (*D*-prunasin and sambunigrin).

**Table 1** Chemical constituents of Xingren decoction, MX decoction, Xingren granules and MX granules by LC-MS.

| Extract | Compound | Content(mg/g) |
| --- | --- | --- |
| Xingren decoction | *D*-amygdalin | 14.7 |
|  | Neoamygdalin | 13.4 |
| MX decoction | *D*-amygdalin | 25.6 |
|  | Neoamygdalin | 9.69 |
| Xingren dispensing granule | *D*-amygdalin | 15.1 |
|  | Neoamygdalin | 16.9 |
| MX dispensing granule | *D*-amygdalin | 16.2 |
|  | Neoamygdalin | 17.1 |


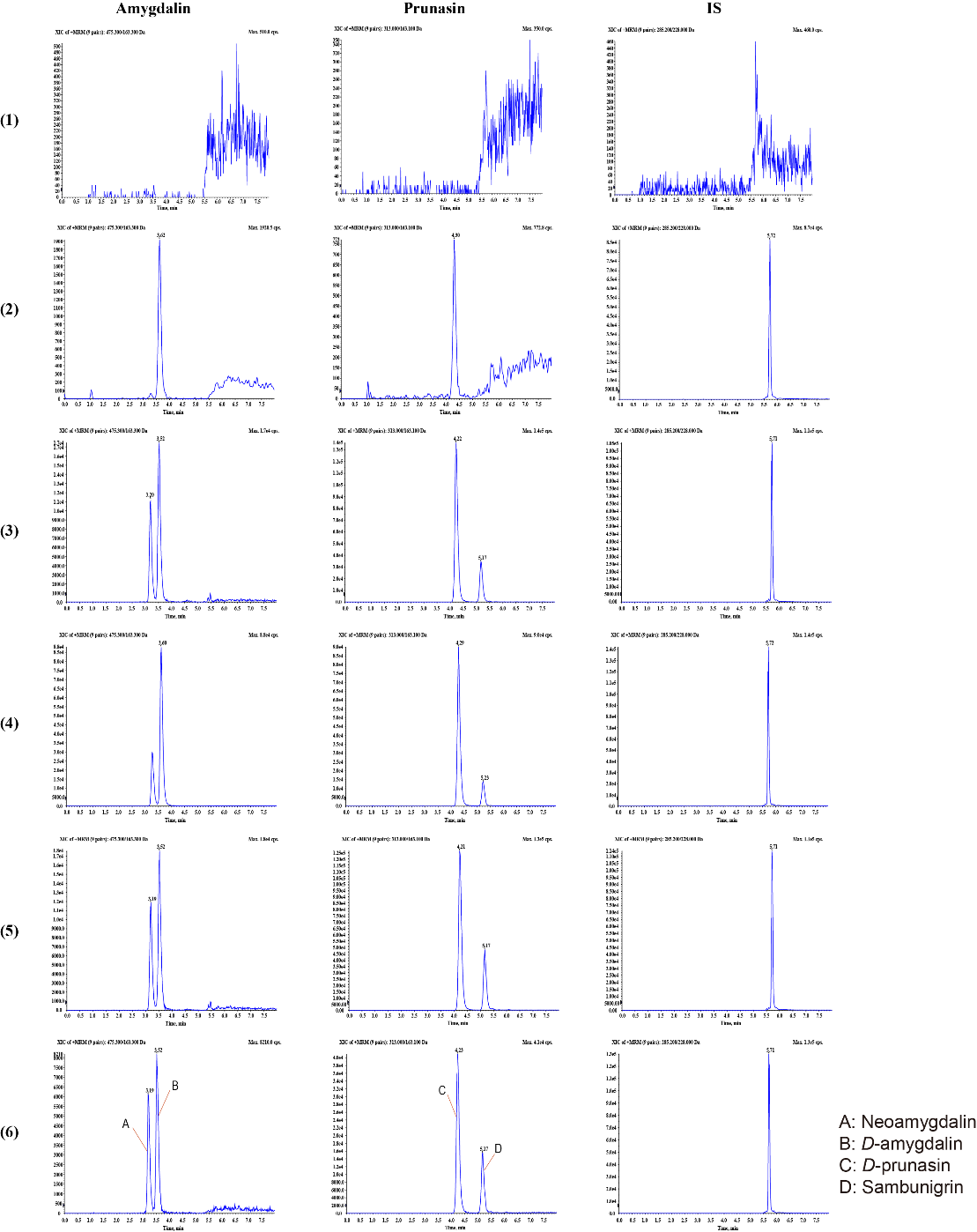


**Figure 3.** Representative multiple reaction monitoring chromatograms of (1) Blank plasma, (2) blank plasma spiked with standard solutions in LLOQ, (3) plasma sample after oral administration of Xingren decoction, (4) plasma sample after oral administration of Mahuang-Xingren (MX) decoction, (5) plasma sample after oral administration of Xingren dispensing granule, (6) plasma sample after oral administration of MX dispensing granule.

**Table 2** Regression data, lower limit of quantiﬁcation (LLOQ) for amygdalin and prunasin.

| Analytes | Regression equation | Correlation coefficient ® | Linear range (ng·mL^-1^) | LLOQ (ng·mL^-1^) |
| --- | --- | --- | --- | --- |
| Amygdalin | *Y*=0.0120x+0.0029 | 0.9969 | 1.00—128.1 | 1.00 |
| Prunasin | *Y*=0.0019x+0.0010 | 0.9993 | 4.62—590.7 | 4.62 |

| Analytes | Concentration (ng·mL^-1^) | Intra-day |  |  | Inter-day |  |  |
| --- | --- | --- | --- | --- | --- | --- | --- |
|  |  | Mean±SD | CV (%) | Accuracy (%) | Mean±SD | CV (%) | Accuracy (%) |
| Amygdalin | 2.20 | 1.99±0.20 | 5.97 | 91.8 | 2.40±0.30 | 12.4 | 109.2 |
|  | 22.0 | 20.2±1.20 | 5.48 | 98.1 | 24.0±0.31 | 1.29 | 109.2 |
|  | 109.8 | 107.7±5.90 | 13.8 | 107.0 | 124.0±10.1 | 8.16 | 113.0 |
| Prunasin | 9.13 | 8.06±0.51 | 6.34 | 88.3 | 10.3±1.37 | 13.3 | 112.9 |
|  | 91.3 | 79.4±4.66 | 5.88 | 86.9 | 99.1±2.34 | 2.36 | 108.5 |
|  | 456.5 | 425.4±20.7 | 4.87 | 93.2 | 519.4±37.2 | 7.17 | 113.8 |
| CV: Coefficient of variation | | | | | | | |

**Table 3** Intra- and inter-day accuracy and precision for amygdalin and prunasin (n=6).

**Table 4** Stability testing of amydalin and prunasin under different conditions of storage in rat plasma (n = 6).

| Analytes | Concentration (ng·mL^-1^) | Short-term (4 h at 25℃) | | 3-freeze-thaw cycles (-20℃ to 4℃) | | Long-term (-40°C, 14 days) | | Post-preparation (24h, 4°C) | |
| --- | --- | --- | --- | --- | --- | --- | --- | --- | --- |
|  |  | Accuracy (%) | CV (%) | Accuracy (%) | CV (%) | Accuracy (%) | CV (%) | Accuracy (%) | CV (%) |
| Amygdalin | 2.20 | 96.3 | 3.01 | 112.6 | 4.39 | 114.7 | 7.82 | 110.9 | 14.3 |
|  | 22.0 | 109.8 | 5.40 | 109.4 | 2.43 | 107.9 | 2.41 | 98.6 | 6.29 |
|  | 109.8 | 114.8 | 4.04 | 113.6 | 2.83 | 113.6 | 3.95 | 108.9 | 6.95 |
| Prunasin | 9.13 | 99.5 | 6.21 | 113.2 | 5.15 | 107.6 | 6.02 | 102.4 | 8.19 |
|  | 91.3 | 110.3 | 7.44 | 106.0 | 2.34 | 108.9 | 1.56 | 94.3 | 5.63 |
|  | 456.5 | 114.5 | 4.85 | 113.1 | 1.56 | 112.3 | 4.26 | 108.3 | 6.73 |
| CV: Coefficient of variation | | | | | | | | | |

**Table 5** Pharmacokinetic parameters of *D*-amygdalin, neoamygdalin, *D*-prunasin and sambunigrin in rats after oral administration of Xingren decoction and dispensing granule.

| Compound | Group | AUC**_0-t_/**Dose  (h·ng·mL^-1^) | C_max_**/**Dose  (ng·mL^-1^) | T_max_  (hour) | t_1/2_  (hour) | CL  (L·h^-1^·kg^-1^) | V_d_  (L·kg^-1^) |
| --- | --- | --- | --- | --- | --- | --- | --- |
| *D*-amygdalin | *Decoction* | 11.46±2.1^**^ | 6.61±2.12^*^ | 0.38±0.14 | 0.85±0.10 | 89.24±17.16^*^ | 109.59±23.33^*^ |
|  | *Dispensing granule* | 4.94±1.24 | 3.74±0.28 | 0.44±0.13 | 0.84±0.19 | 214.21±67.76 | 262.51±117.45 |
| Neoamygdalin | *Decoction* | 1.56±0.32^**^ | 1.18±0.38 | 0.38±0.14 | 0.97±0.25 | 660.25±142.11^*^ | 920.43±266.32 |
|  | *Dispensing granule* | 0.78±0.20 | 0.72±0.11 | 0.50±0.00 | 0.85±0.34 | 1355.38±422.24 | 1649.78±762.94 |
| *D*-prunasin | *Decoction* | 247.91±40.24 | 86.61±15.50 | 1.25±0.50 | 1.50±0.23 | 4.10±0.77 | 8.95±2.73 |
|  | *Dispensing granule* | 213.71±23.68 | 95.74±7.24 | 1.25±0.29 | 1.56±0.18 | 4.70±0.57 | 10.53±1.57 |
| Sambunigrin | *Decoction* | 23.80±2.08^**^ | 10.50±1.18^*^ | 0.81±0.24 | 1.33±0.25 | 42.13±3.98^**^ | 81.32±22.14^*^ |
|  | *Dispensing granule* | 15.23±1.28 | 8.03±0.94 | 0.69±0.13 | 1.29±0.27 | 65.81±5.71 | 121.44±24.13 |
| ^*^ *P* < 0.05, ^**^ *P* < 0.01 *vs. decoction*. | | | |  |  |  |  |

**Table 6** Pharmacokinetic parameters of *D*-amygdalin, neoamygdalin, *D*-prunasin and sambunigrin in rats after oral administration of MX decoction and dispensing granule.

| Compound | Group | AUC**_0-t_/**Dose  (h·ng·mL^-1^) | C_max_**/**Dose  (ng·mL^-1^) | T_max_  (hour) | t_1/2_  (hour) | CL  (L·h^-1^·kg^-1^) | V_d_  (L·kg^-1^) |
| --- | --- | --- | --- | --- | --- | --- | --- |
| *D*-amygdalin | *Decoction* | 31.39±4.60^**^ | 24.11±4.71^**^ | 0.50±0.20 | 1.39±0.92 | 32.19±4.09^**^ | 76.34±28.44^*^ |
|  | *Dispensing granule* | 4.69±0.62 | 3.63±0.33 | 0.46±0.28 | 0.59±0.27 | 215.65±30.62 | 182.08±54.12 |
| Neoamygdalin | *Decoction* | 5.77±2.43 | 6.22±2.39^**^ | 0.50±0.20 | 0.97±0.33 | 224.84±134.24^**^ | 315.35±121.87^*^ |
|  | *Dispensing granule* | 0.70±0.08 | 0.64±0.10 | 0.46±0.28 | 0.62±0.27 | 1438.09±146.74 | 1312.94±679.68 |
| *D*-prunasin | *Decoction* | 355.39±50.39^*^ | 163.11±32.69 | 1.13±0.25 | 1.82±0.58 | 2.83±0.44^*^ | 7.25±1.71 |
|  | *Dispensing granule* | 278.90±33.00 | 183.14±36.83 | 0.81±0.13 | 1.74±0.55 | 3.61±0.45 | 8.83±1.86 |
| Sambunigrin | *Decoction* | 24.53±6.63 | 13.94±2.12 | 0.81±0.13 | 1.12±0.34 | 42.53±10.32 | 65.62±8.45 |
|  | *Dispensing granule* | 20.15±2.57 | 15.78±2.51 | 0.75±0.00 | 0.88±0.09 | 49.70±5.83 | 63.42±12.15 |
| ^*^ *P* < 0.05, ^**^ *P* < 0.01 *vs. decoction*. | | | | |  |  |  |

**Table 7** Pharmacokinetic parameters of ratio of *D*-amygdalin to neoamygdalin in rats after oral administration of decoction and dispensing granule.

| Group | AUC**_0-t_/**Dose | C_max_**/**Dose | V_d_ | CL |
| --- | --- | --- | --- | --- |
| Xingren decoction | 7.19±0.25 | 5.19±0.46 | 0.12±0.04 | 0.13±0.01 |
| Mahuang-Xingren decoction | 5.72±0.87^*^ | 4.9±0.39 | 0.2±0.03 | 0.19±0.03^*^ |
| Xingren dispensing granule | 6.12±0.38 | 6.12±0.38 | 0.16±0.02 | 0.16±0.01 |
| Mahuang-Xingren dispensing granule | 6.25±1.21 | 6.25±1.12 | 0.19±0.01 | 0.16±0.04 |
| ^*^ *P* < 0.05, ^**^ *P* < 0.01 *vs.* *Mahuang-Xingren* | | | | |

**Table 8** Metabolic stability data of amygdalin in RLM and RGME

| Parameter | RLM | RGME |
| --- | --- | --- |
| t_1/2_(min) | 27.38±3.77 | 13.16±1.65 |
| CL_int_ (uL/min/mg protein) | 25.66±3.31 | 53.36±7.49 |


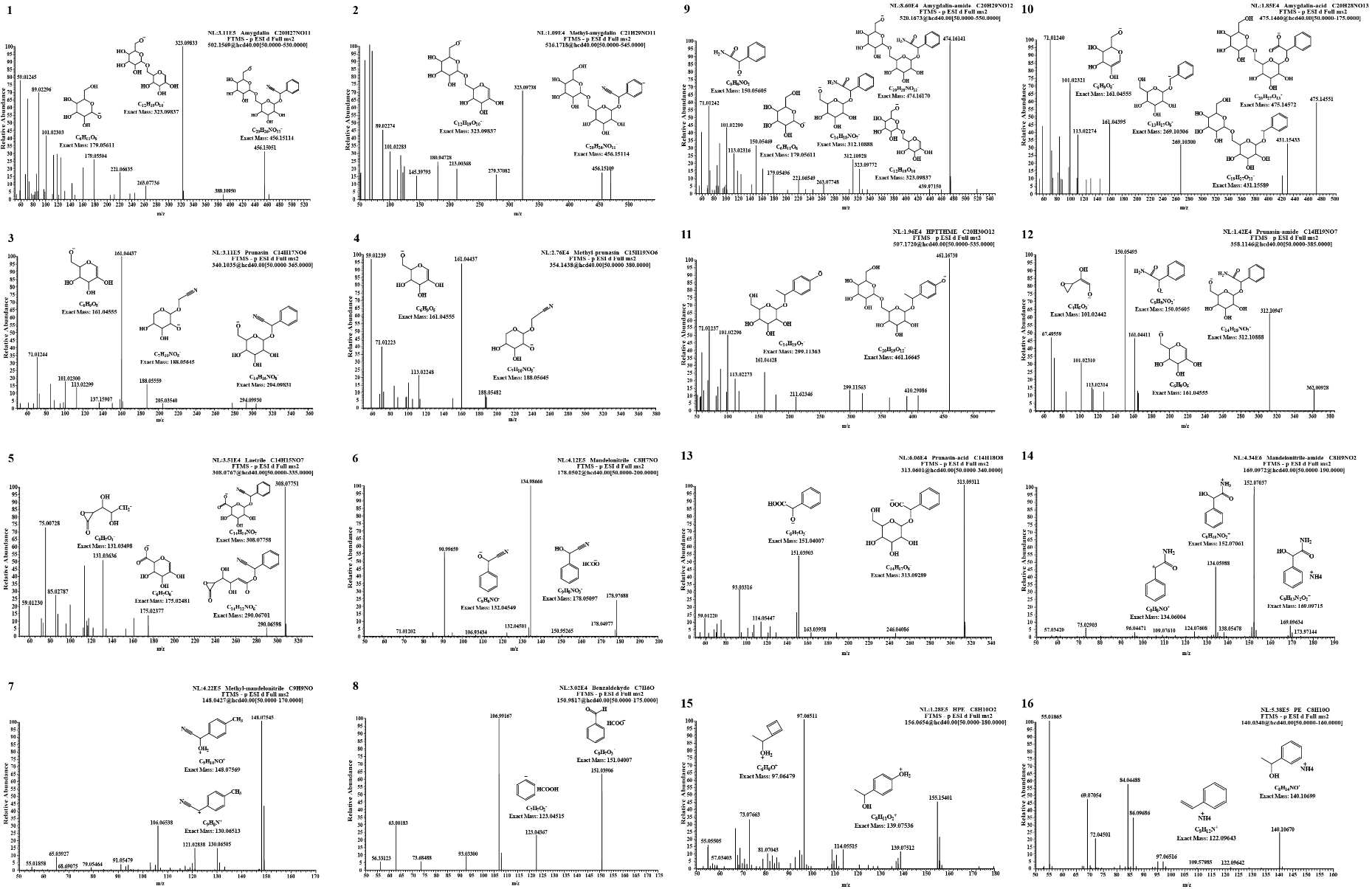


**Figure 4.** Fragmentation mass spectra of 16 compounds with purposed fragmentation pathways.


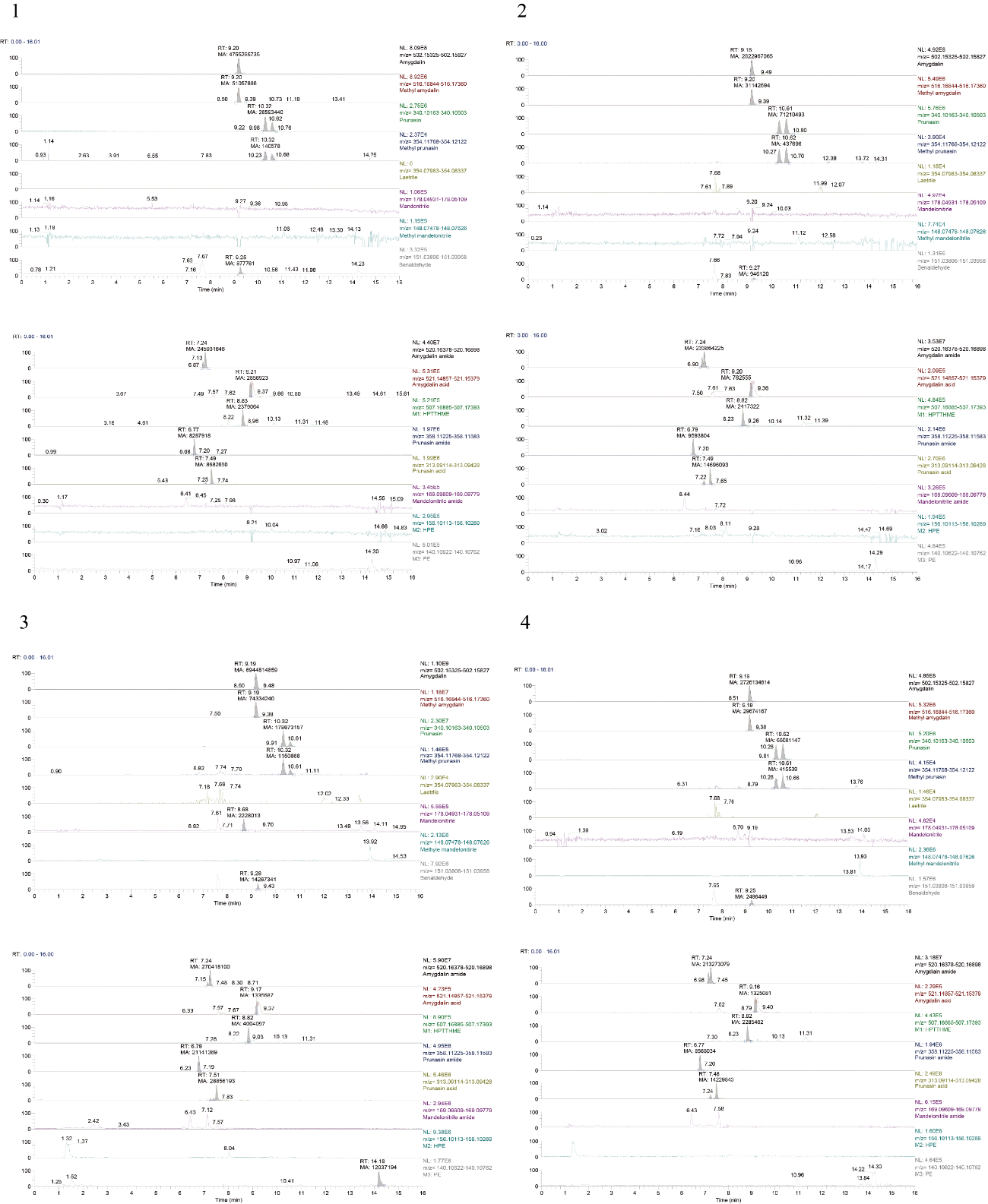


**Figure 5.** Extracted ion chromatogram of (1) *Xingren* decoction, (2) *Mahuang-Xingren* decoction, (3) *Xingren* dispensing granule, (4) *Mahuang-Xingren* dispensing granule.
